# Child-Report Measures of Occupational Performance: A Systematic Review

**DOI:** 10.1371/journal.pone.0147751

**Published:** 2016-01-25

**Authors:** Reinie Cordier, Yu-Wei Chen, Renée Speyer, Rebekah Totino, Kenji Doma, Anthony Leicht, Nicole Brown, Belinda Cuomo

**Affiliations:** 1 School of Occupational Therapy and Social Work, Curtin University, Perth, WA, Australia; 2 Faculty of Health Sciences, The University of Sydney, Sydney, NSW, Australia; 3 College of Healthcare Sciences, James Cook University, Townsville, QLD, Australia; University of Rochester, UNITED STATES

## Abstract

**Introduction:**

Improving occupational performance is a key service of occupational therapists and client-centred approach to care is central to clinical practice. As such it is important to comprehensively evaluate the quality of psychometric properties reported across measures of occupational performance; in order to guide assessment and treatment planning.

**Objective:**

To systematically review the literature on the psychometric properties of child-report measures of occupational performance for children ages 2–18 years.

**Methods:**

A systematic search of the following six electronic databases was conducted: CINAHL; PsycINFO; EMBASE; PubMed; the Health and Psychosocial Instruments (HAPI) database; and Google Scholar. The quality of the studies was evaluated against the COSMIN taxonomy of measurement properties and the overall quality of psychometric properties was evaluated using pre-set psychometric criteria.

**Results:**

Fifteen articles and one manual were reviewed to assess the psychometric properties of the six measures–the PEGS, MMD, CAPE, PAC, COSA, and OSA- which met the inclusion criteria. Most of the measures had conducted good quality studies to evaluate the psychometric properties of measures (PEGS, CAPE, PAC, OSA); however, the quality of the studies for two of these measures was relatively weak (MMD, COSA). When integrating the quality of the psychometric properties of the measures with the quality of the studies, the PAC stood out as having superior psychometric qualities.

**Conclusions:**

The overall quality of the psychometric properties of most measures was limited. There is a need for continuing research into the psychometric properties of child-report measures of occupational performance, and to revise and improve the psychometric properties of existing measures.

## Introduction

Occupations can be comprehensively defined as the ‘…groups of activities and tasks of everyday life, named, organized, and given value and meaning by individuals and a culture. Occupation is everything people do to occupy themselves, including looking after themselves (self-care), enjoying life (leisure) and contributing to the social and economic fabric of their communities (productivity)’[1] (p.34). Occupational performance, the act of *doing* an occupation in order to satisfy life’s needs [2], is an important construct across the lifespan. Conceptually, occupational performance is the point at which the person, the environment and the occupation–in a dynamic interplay—support the tasks, activities and roles that define that person as an *individual* [1, 2]. When people have difficulty in performing occupations, the role of the occupational therapist is to work with the person to improve their performance (e.g., maintaining upper limb strength to use a wheelchair for a person with a spinal cord injury [SCI]), to adapt the materials they use to perform the occupations (e.g., prescribe a wheelchair for a person with SCI) and to recommend changes to the environment (e.g., fitting wheelchair ramps around the house for a person with SCI) [1]. As occupational performance is necessary for daily life, occupational performance assessments are integral to the delivery of health services to clients, particularly by occupational therapists. In order to support clients in building towards optimal performance, occupational therapists must have access to assessments that enable consideration of all of the occupations of individuals in their daily lives [2] as being sensitive to their specific needs, experiences, and expectations [3].

Gaining an understanding of a person’s perspective in treatment is important to client-centred practice, which aims to actively involve the client in treatment plans, and respond to the client’s knowledge of their own needs [4]. In recognition of this, clinical practice has recently transitioned to a family-centred model where both the adult and the child is actively involved in the treatment process [5]. Involving children in the intervention process has been shown to improve treatment outcomes [6]. Furthermore, children’s perceptions affect their quality of life, so collecting information from the child’s perspective is essential in clinical practice, in order to meet the aim of improving quality of life for the child [7, 8].

Currently, there is a paucity of systematic research that evaluates the psychometric properties of child-report measures of occupational performance. Given that the child’s perspective is important when gathering information used to guide clinical practice and client- and/or family-centred occupational practice, systematically reviewing the psychometric properties of existing child-report measures of such performance are integral to evidence-based practice.

### Assessments of Occupational Performance

Descriptive tools, which measure clients’ current functional status, problems, needs, or circumstances [9], are often employed to measure occupational performance, and are regarded to best fit with client-centred occupational therapy practice. Self-report measures are a descriptive tool commonly associated with the client centred approach to practice [10] and are useful in determining the client’s *own* perspective of performance and experience [2]. Self-report measures vary in what aspect of occupational performance they measure, specifically “capacity” versus “actual” performance [2]. However, it is important to note that there are difficulties in accurately determining “true” performance due to over- or under-estimation of performance by the individual. Subsequently, systematic reviews of available measures are integral to guiding selection of the most appropriate available self-report measures.

### Assessments of Occupational Performance and Children

Commonly, childhood assessments of occupational performance rely on developmental tests that are based on the assumption that normalising processes, such as occupational performance, are integral to achieving better functioning [11]. The tools employed in the client-centred approach for occupational performance assessments should capture children’s perceptions of their strengths and capabilities of their daily activities, rather than solely focusing on impairment [12, 13]. Occupational performance assessments should include identification of the child’s occupations, what occupations are motivating and important, and the compatibility between characteristics of the child and their environment to create successful occupational performance [12, 13].

There can be challenges when adopting a client-centred approach for assessment of occupational performance [3]. There are differing views around who should be the focus of assessment when the client is a child, as children are commonly in environments where the standards and expectations are set by others (e.g., at school by teachers) [3]. Therefore, there is uncertainty over how the child will be able to determine his or her needs and goals relating to occupational performance. As a consequence, many “self-report” instruments measuring occupational performance of children are in fact teacher- or parent-reports [14]. There is a need, however, to incorporate measures that are child-based, in order to gather data that is meaningful to the *child*. Additionally, occupational therapists and other allied health professionals have a duty to choose self-report measures that have established validity, reliability and clinical utility in order to inform holistic interventions for the child [13, 15].

### Reviews of Occupational Performance

Currently, there have been numerous systematic reviews of the psychometrics of various instruments for measuring specific occupational performance components, which include children in the inclusion criteria [e.g., 16, 17–25]. Of these systematic reviews, many focus on measures of occupational performance for use with specific diagnostic groups of children, for example Acquired Brain Injury (ABI) or Cerebral Palsy (CP) [12, 13, 16, 24–28].

#### Participation and Activity

Under the International Classification of Functioning (ICF), Disability and Health, activity is defined as the ‘execution of a task or action by an individual’, while participation refers more broadly to ‘involvement in a life situation’ [29]. The promotion of health and wellbeing by enabling participation in occupations within a rehabilitation context is a typical goal in occupational therapy; participation measures are therefore commonly used when assessing occupational performance. Many systematic reviews of diagnostic groups have focused on measures of participation domains, commonly following the International Classification of Functioning, Disability and Health–Children and Youth (ICF-CY) domains of activity and participation, such as learning and applying knowledge, general tasks and demands, mobility, self-care and major life areas [30]. [19] reported in their systematic review that measures covered all ICF-CY domains of participation and activity for children with ABI, and that self-care in particular was covered well. The authors concluded that the occupational therapy assessments were more holistic in occupational performance, unlike medical assessments which were commonly related with bodily functions [19].

#### Self-care

A 2012 review focused on self-care as a specific domain of participation and activity. Ireland and Johnston (21)[21] systematically reviewed the validity, clinical utility, and reliability of measures which evaluated the self-care skills of children (0–12 years) with the congenital musculoskeletal condition of osteochondrodysplasia. The authors found that the available measures (Functional Independence Measure for Children (WeeFIM), the Activities Scale for Kids (ASK), and the Pediatric Evaluation of Disability Inventory (PEDI) ranged from adequate to excellent in reliability, and that there was evidence of validity, for the particular diagnostic group. This review indicates that assessments of self-care which employ these measures can give, in the least, an adequate understanding of children from this diagnostic groups’ level of self-care.

#### Occupational Performance

Some systematic reviews have focused on the broader domain of occupational performance. For example, Parker and Sykes (31)[31] systematically assessed studies that examined the effects of outcome measures of the Canadian Occupational Performance Measure (COPM) on clinical occupational therapy practice using thematic analysis. The authors concluded that the COPM had the greatest impact within clinical practice, and that further research into other clinical areas as well as the need for more training in using the COPM as an outcome measure was needed [31]. Whilst this review is important in terms of clinical implications, the analysis is qualitative, and thus, does not shed light on the psychometric properties of the COPM.

These reviews have focused on the relevance of instruments measuring occupational performance in the context of these specific diagnostic groups and their needs. However, there is a notable lack of systematic reviews that focus on self-report measures used to assess occupational performance in children [32].

### Study Aim

There is still a paucity of systematic reviews on child-report measures of occupational performance despite it being a key service for occupational therapists. Subsequently, the purpose of this systematic review is to identify instruments that measure occupational performance in children through child-report methods, and to appraise the psychometric properties of these measures. This systematic review focuses on the psychometric properties of instruments used by occupational therapists for samples of children 2–18 years, written in English. The COSMIN taxonomy of measurement properties and definitions for health-related patient-reported outcomes was used to evaluate each instrument in the domains of reliability, validity and responsiveness [33]. COSMIN aims to improve the selection of health measurement instruments by providing a checklist on methodological qualities of the tools [34]. Consideration of responsiveness, the ability of a measure to detect change in a construct over time [34], was deemed to be outside the scope of this review. Evaluating responsiveness as a psychometric property involves assessing all articles that used the included assessments as outcome measures. Given that including responsiveness would increase the size of this systematic review exponentially, we are of the opinion that an investigation of this property warrants a separate and more detailed systematic review. It is expected that this systematic review will assist in the choice of instruments measuring occupational performance, by providing an objective account of the advantages and disadvantages of self-report measures available for children.

## Methods

Methodology and writing of the systematic review was guided by the use of the PRISMA statement [35]. The PRISMA statement is a checklist comprised of 27 item areas that are considered to be crucial for ensuring transparency when conducting systematic reviews. Please refer to S1 Table for the completed PRISMA checklist for the current review.

### Eligibility Criteria

Studies deemed eligible for inclusion included both research articles and published manuals that detailed the psychometric properties of instruments designed to measure the occupational performance of children. Occupational performance assessments should include identification of a child’s occupations, what occupations are motivating and important, and how the characteristics of that person combine with the environment in which the occupation occurs to create successful occupational performance [10, 12, 36]. Within this search, self-report instruments that measured skills and behaviours relating to occupational performance in children (2–18 years) were included. To be included, abstracts and instruments additionally needed to be primarily designed for use with children, be used by occupational therapists, and written in English. Articles and instruments were excluded if infants or adults were included in the sample, the selected instrument was not the main focus (i.e., another instrument was using the selected instrument for construct validity), and the full text was unable to be retrieved. Dissertations and conference papers were excluded.

### Information Sources

A preliminary systematic literature search was performed on 25^th^ April, 2014 by two authors using the following four electronic databases: Embase; PubMed; PsycINFO; and CINAHL. Both subject headings and free text were used when searching each database. Date restrictions were imposed in free text searching. See Table 1 for a complete list of search terms used across all searches. A second literature search on 7^th^ July 2014 using the title of the instrument and its acronym was conducted in CINAHL, PsycInfo, EMBASE, and PubMed (see Table 1 for search terms) to identify psychometric articles. Additionally, manuals for the instruments were retrieved for appraisal. A third literature search in Google Scholar and EBSCO Host’s Health and Psychological Instruments (HAPI) database using the title of the instrument and its acronym was conducted by two research assistants from 11^th^ November 2014 to 17^th^ November 2014. The aim of the search in Google Scholar was to identify any recently published articles (Publication year: 2013–2014). The HAPI database was used for more specific searching and results (see Table 1 for search terms used).

**Table 1:** Search Terms.

**Initial search: Assessment retrieval Database and Search Terms using Subject Headings**	**Limitations**
**CINAHL**: (((Outcome assessment) OR (Patient assessment) OR (Clinical assessment tools) OR (Occupational therapy assessment) OR (Functional assessment) OR (Self-assessment) OR (Functional assessment inventory) OR (Neurologic examination)) OR (Psychological Tests) OR (Questionnaires) OR ((Severity of Illness Indices) OR (Diagnosis, Developmental) OR (Diagnosis, Psychosocial) OR (Disability evaluation)) OR ((Scales) OR (Behaviour Rating Scales)) OR ((Health screening) OR (Health screening Iowa NIC) OR (Denver Developmental Screening Test)) OR ((Treatment Outcomes) OR (Evaluation)) AND ((Occupational therapy) OR (Paediatric occupational therapy) OR (Occupational therapy practice, research-based) OR (Occupational therapy practice, evidence-based))	English Language; Human, Preschool age (2–5 years), School Age (6–12 years), Adolescence (13–17 years)
**PsycINFO**: (measurement) OR (Psychological assessment)) or (Cognitive assessment)) OR (Questionnaires)) OR (Neuropsychological assessment)) OR (Testing)) OR (Testing methods)) OR (Rating scales)) OR (Screening OR Screening tests) OR (Treatment outcomes)) OR (Evaluation) AND (Occupational therapy)	English language; Human, Preschool age (2–5 years), School Age (6–12 years), Adolescence (13–17 years
**Embase:** ((Disability) OR (Measurement) OR (Questionnaire) OR (Rating scale) OR (Screening) OR (Outcome assessment) OR (Evaluation study)OR (Occupational therapy assessment)) AND (Occupational therapy)	English language; Human, Preschool age (2–5 years), School Age (6–12 years), Adolescence (13–17 years),
**PubMed:** (((((((("Outcome and Process Assessment (Health Care)"[Mesh] OR "Patient Outcome Assessment"[Mesh] OR "Symptom Assessment"[Mesh] OR "Outcome Assessment (Health Care)"[Mesh] OR "Self-Assessment"[Mesh])) OR (("Severity of Illness Index"[Mesh]) OR "Health Status Indicators"[Mesh])) OR "Questionnaires"[Mesh]) OR (("Neuropsychological Tests"[Mesh]) OR ("Psychological Tests"[Mesh] OR "Intelligence Tests"[Mesh]))) OR "Outcome Assessment (Health Care)"[Mesh]) OR (("Program Evaluation"[Mesh] AND "Diagnostic Self Evaluation"[Mesh] AND "Disability Evaluation"[Mesh] AND "Symptom Assessment"[Mesh]) OR "Treatment Outcome"[Mesh]))) AND ("Occupational Therapy"[Mesh] OR "Occupational Therapy Department, Hospital"[Mesh])	English language; Human, birth-18 years,
**Initial search: Assessment retrieval Database and Search Terms using Free Text Words**	**Limitations**
**CINAHL:** (assessment* OR measure* OR questionnaire* OR test OR test* OR scale* OR screening* OR evaluation*) AND (occupational)	English, Preschool age (2–5 years), School Age (6–12 years), Adolescence (13–17 years), human, Publication year: 20130401–20140531
**PsycINFO:** *As per CINAHL Free Text*	English, Preschool age (2–5 years), School Age (6–12 years), Adolescence (13–17 years), human, Publication year 2013–2014
**Embase:** *As per CINAHL Free Text*	English, Preschool age (<1–5 years), School Age (6–12 years), Adolescence (13–17 years), human, Publication year 2013–2014
**PubMed:** *As per CINAHL Free Text*	English, Child: birth-18 years, human, Publication year 20130425–20141231
**Second search: Pyschometric articles Database and Search Terms using Subject Headings**	**Limitations**
**CINAHL**: (MH "Validity") OR (MH "Criterion-Related Validity") OR (MH "Predictive Validity") OR (MH "Reliability and Validity") OR (MH "Internal Validity") OR (MH "External Validity") OR (MH "Face Validity") OR (MH "Construct Validity") OR (MH "Content Validity") OR (MH "Discriminant Validity") OR (MH "Consensual Validity") OR (MH "Concurrent Validity") OR (MH "Qualitative Validity") (MH "Interrater Reliability") OR (MH "Intrarater Reliability") OR (MH "Reliability and Validity") OR (MH "Reliability") OR (MH "Psychometrics") OR (MH "Instrument Validation") AND (“Name of instrument” OR “Acronym of instrument))	English Language; Human
**PsycINFO**: (Psychometrics/ OR statistical reliability/ OR statistical validity/ OR “error of measurement”/) AND (“Name of instrument” OR “Acronym of instrument))	English language; Human
**Embase:** (Validation study/ OR validity/ OR psychometry/ OR reliability/ OR measurement accuracy/ OR measurement error/ OR measurement precision/OR measurement repeatability/) AND (“Name of instrument” OR “Acronym of instrument))	English language; Human
**PubMed:** ("Reproducibility of Results"[Mesh]) OR ("Validation Studies" [Publication Type] OR "Validation Studies as Topic"[Mesh])) OR "Psychometrics"[Mesh]) AND (“Name of instrument” OR “Acronym of instrument))	English language; Human
**Third search: Pyschometric articles Database and Search Terms using Free Text Words**	**Limitations**
**Google Scholar:** ("Psychometrics" OR "Measurement" OR "Test Construction" AND “instrument name (ACRONYM)” AND “child report”) (reliability" OR "psychometry" OR "validity" OR "validation study" OR "instrument validation" AND " instrument name (ACRONYM)" AND “child report”)	Publication year: 2013–2014
**Third search: Pyschometric articles Database and Search Terms using Subject Headings**	**Limitations**
**HAPI:** ("Psychometrics" OR "Measurement" OR "Test Construction" AND “instrument name (ACRONYM)” AND “child report”) (reliability" OR "psychometry" OR "validity" OR "validation study" OR "instrument validation" AND " instrument name (ACRONYM)" AND “child report”)	None

### Study Selection

**Fig 1:**
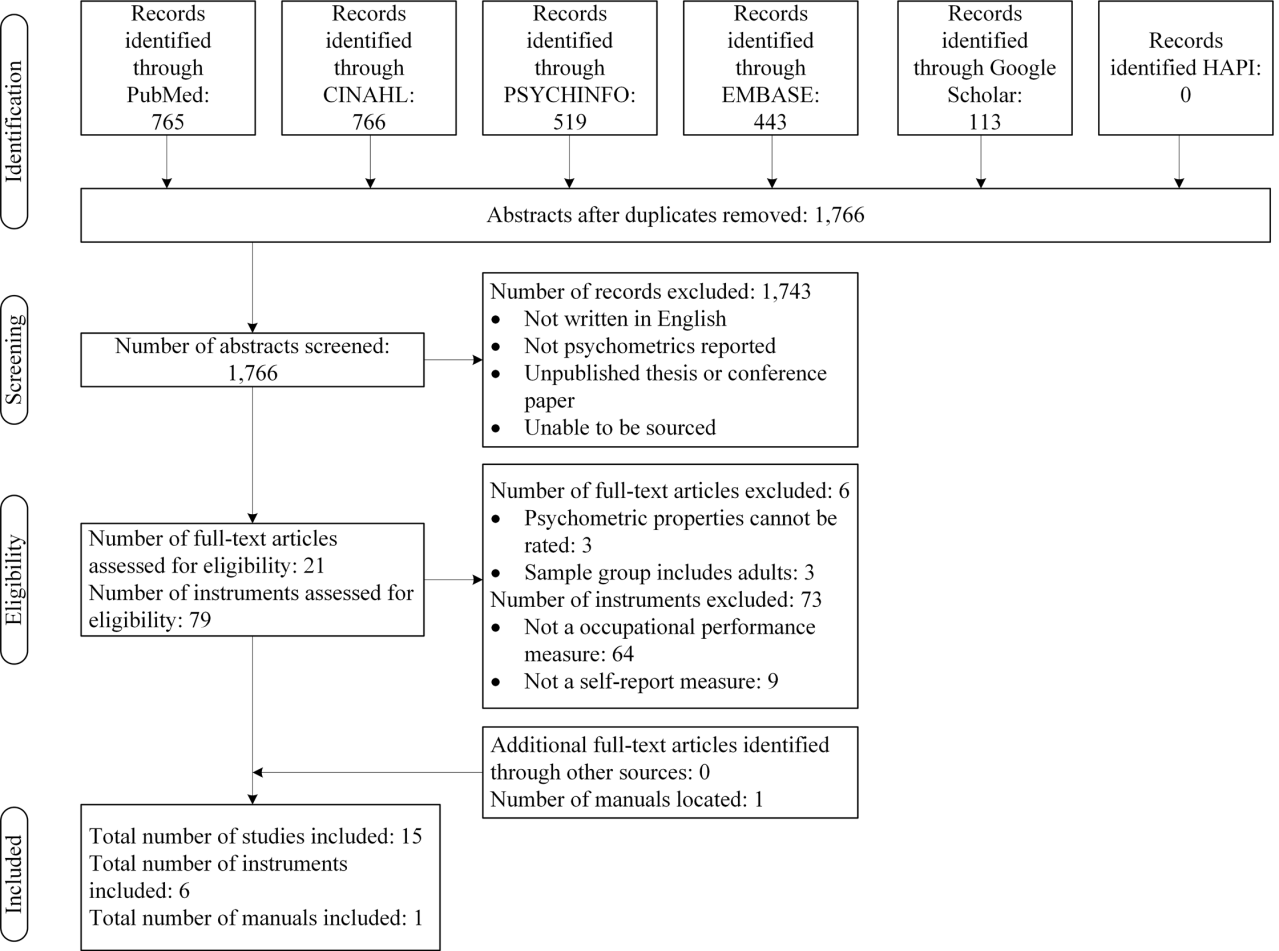
Flowchart of included studies, manuals and measures.

All abstracts were rated by a reviewer on the following inclusion criteria: the measure had to assess occupational performance; abstracts had to contain a child-based tool; the main target group of the instrument was children; and it needed to be used by occupational therapists. The names of instruments were retrieved from the identified abstracts. A flowchart depicting this process is shown in Fig 1. To determine the inter-rater reliability scores between both reviewers, a random sample of 40% of the abstracts was used. The interrater reliability between raters were deemed acceptable: Weighted Kappa = 0.72.

### Data Collection Process and Data Extraction

First, data from studies and manuals for the development and validation of occupational performance assessment instruments were extracted under the following descriptive categories: study design, purpose of the study, study population, age of the population, and instrument characteristics. Additionally, the COSMIN [33] criteria were used to assess the methodological quality of the studies.

### Methodological Quality

The COSMIN taxonomy of measurement properties and definitions for health-related patient-reported outcomes were used to assess the methodological quality of the included studies [33, 34]. The COSMIN checklist contains nine domains: internal consistency, reliability (relative measures: including test-retest reliability, inter-rater reliability and intra-rater reliability), measurement error (absolute measures), content validity (including face validity), structural validity, hypotheses testing, cross-cultural validity, criterion validity, and responsiveness. As interpretability was not considered to be a psychometric property, it was not included in this review. Definitions of each of the measurement properties of the COSMIN are shown in Table 2.

**Table 2:** COSMIN: Definitions of domains, psychometric properties, and aspects of psychometric properties for Health-Related Patient-Reported Outcomes (adapted from Mokkink, Terwee [42]).

Psychometric property	Domain: Definition^a^
	**Validity:** the extent to which an instrument measures the construct/s it claims to measure.
**Content validity**	The degree that the content of an instrument adequately reflects the construct to be measured.
Face validity^b^	The degree to which instrument (items) appear to be an adequate reflection of the construct to be measured.
**Construct validity**	The extent to which the scores of an instrument are consistent with hypotheses, based on the assumption that the instrument is a valid measure of the construct being measured.
Structural validity^c^	The extent to which instrument scores adequately reflect the dimensionality of the construct to be measured.
Hypothesis testing^c^	Item construct validity.
Cross-cultural validity^c^	The extent that performance of the items on a translated or culturally adapted instrument adequately replicates the performance of the items of the original version of the instrument.
**Criterion validity**	The degree to which the scores of an instrument satisfactorily reflect a “gold standard”.
**Responsiveness**	**Responsiveness:** the capability of an HR-PRO instrument to detect change in the construct to be measured over time.
**Interpretability**^d^	**Interpretability**^a^: the extent to which qualitative meaning can be given to an instrument’s quantitative scores or score change.
	**Reliability:** the extent to which the measure is free from measurement error.
**Internal consistency**	The level of correlation amongst items.
**Reliability**	The proportion of total variance in the measurements due to “true” differences amongst patients.
**Measurement error**	The error of a patient’s score, systematic and random, *not* attributed to true changes in the construct measured.

Notes.

^a^Applies to Health-Related Patient-Reported Outcomes (HR-PRO) instruments.

^b^Aspect of content validity under the domain of validity.

^c^Aspects of construct validity under the domain of validity.

^d^Interpretability is not considered a psychometric property

Each COSMIN checklist domain is comprised of between 5 and 18 items, which relate to different aspects of study design and methods of statistical analysis. Scores are obtained via the methods proposed by Terwee et al. [37]. Items are rated on a 4-point scale (excellent, good, fair, and poor), with an overall methodological quality score for each psychometric property calculated from the lowest-rated item in each domain. However, strict adherence to this rating system appears to inhibit differentiation between more subtle psychometric qualities of assessments [38], and therefore for this review a revised scoring was introduced [39]. The outcome was presented as a percentage of rating (Poor = 0–25.0%, Fair = 25.1%-50.0%, Good = 50.1%-75.0%, Excellent = 75.1%-100.0%). To take account of those COSMIN items that do not have all the four response options available, the following formula was used to calculate the total score for each psychometric property in order to most accurately capture the quality of the psychometric properties:

Totalscoreforpsychometricproperty=(Totalscoreobtained−minimalpossiblescore)÷(Maximumscorepossible−minimumscorepossible)×100

For example, the COSMIN psychometric property *content validity* has 5 items to be scored. The following scores are allocated: poor = 1, fair = 2, good = 3 and excellent = 4. For 1 of the 5 items, poor as a rating is not possible, but all items have excellent as a rating option. Thus the minimum score possible for this property is 6 (4 items x score of 1 + 1 item x score of 2 = 6) and the maximum possible score is 20 (5 items x score of 4). So for example if a measure received a score of 15 for content validity, the total score for the psychometric property was calculated as (15–6)/ (20–6) x 100 = 64.3%, which classifies content validity as having good quality.

To ensure consistency on the COSMIN checklist ratings, training of an additional rater was completed by the second author who has extensive experience in the area and also was one of the raters. Both authors scored all the papers; consensus was reached where there were differences in ratings. The first author helped resolved differences in ratings where consensus could not be reached between the two raters.

Once the *quality of the studies* that examined the psychometric properties were assessed using the COSMIN system, the actual *quality of the psychometric properties* of the measures reported were evaluated using criteria set out by Terwee et al. [40]. Table 3 provides a summary of the criteria for rating the psychometric quality of the measures. Studies that received a poor COSMIN rating were excluded from further analysis and was awarded a score of NE (Not evaluated). Finally an overall quality score for each measurement property for all assessments was determined using the criteria introduced by Schellingerhout et al. [41], which integrates the scores from the COSMIN ratings with the psychometric quality ratings by Terwee et al. [40], thus generating an overall quality rating.

**Table 3:** Criteria of psychometric quality rating (adapted from Terwee et al. [40]).

Psychometric property	Score^a^	Quality Criteria^b^
**Content validity**	+	A clear description is provided of the measurement aim, the target population, the concepts that are being measured, and the item selection AND target population and (investigators OR experts) were involved in item selection
	?	A clear description of above-mentioned aspects is lacking OR only target population involved OR doubtful design or method
	-	No target population involvement
	±	Conflicting results
	NR	No information found on target population involvement
	NE	Not evaluated
**Structural validity**^c^	+	Factors should explain at least 50% of the variance
	?	Explained variance not mentioned
	-	Factors explain < 50% of the variance
	±	Conflicting results
	NR	No information found on structural validity
	NE	Not evaluated
**Hypothesis testing**^c^	+	Specific hypotheses were formulated AND at least 75% of the results are in accordance with these hypotheses
	?	Doubtful design or method (e.g., no hypotheses)
	-	Less than 75% of hypotheses were confirmed, despite adequate design and methods
	±	Conflicting results between studies within the same manual
	NR	No information found on hypotheses testing
	NE	Not evaluated
**Criterion validity**	+	Convincing arguments that gold standard is “gold” AND correlation with gold standard ≥0.70
	?	No convincing arguments that gold standard is “gold” OR doubtful design or method
	-	Correlation with gold standard <0.70, despite adequate design and method
	±	Conflicting results
	NR	No information found on criterion validity
	NE	Not evaluated
**Internal consistency**	+	Factor analyses performed on adequate sample size (7 * # items and ≥ 100) ANDCronbach’s alpha(s) calculated per dimension AND Cronbach’s alpha(s) between 0.70 and 0.95
	?	No factor analysis OR doubtful design or method
	-	Cronbach’s alpha(s) <0.70 or >0.95, despite adequate design and method
	±	Conflicting results
	NR	No information found on internal consistency
	NE	Not evaluated
**Reliability**	+	ICC or weighted Kappa ≥ 0.70
	?	Doubtful design or method (e.g., time interval not mentioned)
	-	ICC or weighted Kappa < 0.70, despite adequate design and method
	±	Conflicting results
	NR	No information found on reliability
	NE	Not evaluated
**Measurement error**^d^	+	MIC < SDC OR MIC outside the LOA OR convincing arguments that agreement is acceptable
	?	Doubtful design or method OR (MIC not defined AND no convincing arguments that agreement is acceptable)
	-	MIC ≥ SDC OR MIC equals or inside LOA, despite adequate design and method;
	±	Conflicting results
	NR	No information found on measurement error
	NE	Not evaluated

Notes.

^a^Scores: + = positive rating,? = indeterminate rating,— = negative rating, ± = conflicting data, NR = not reported, NE = not evaluated (for study of poor methodological quality according to COSMIN rating [Table 6], data are excluded from further evaluation [Tables 7 and 8])

^b^Doubtful design or method is assigned when a clear description of the design or methods of the study is lacking, sample size smaller than 50 subjects (should be at least 50 in every subgroup analysis), or any important methodological weakness in the design or execution of the study

^c^Hypothesis testing: all correlations should be statistically significant (if not, these hypotheses are not confirmed) AND these correlations should be at least moderate (*r* > 0.5)

^d^Measurement error: MIC = minimal important change, SDC = smallest detectable change, LOA = limits of agreement.

### Data Items, Risk of Bias and Synthesis of Results

All data items for each measure were obtained. ‘NR’ was recorded for items that were not reported. Inclusion of ‘methodological limitations items’ during the rating of the COSMIN checklist enabled assessment of risk of bias at an individual study level. The results were extracted and grouped under the following headers: 1) purpose of instrument, 2) year published, and 3) the instrument characteristics.

## Results

### Systematic Literature Search

Following the removal of duplicate abstracts across six databases, a total of 79 measures were reviewed. Of these 79 measures, 73 were excluded for the following reasons: they were not measures of occupational performance (*n* = 64) and they were not self-report measures by children (*n* = 9). Thus, 6 measures met the inclusion criteria. Systematic searches across six databases retrieved 1,766 article abstracts, which were screened for inclusion in this review. Of these articles, 21 full-text articles were assessed for eligibility; 3 articles were excluded as the psychometric properties could not be rated and 3 were excluded as adults were included in the measurement development and testing (see Fig 1 for full details). One manual was located through the secondary (using the name of the identified measures) and tertiary searches (using Google Scholar and HAPI databases—see Table 1 and Fig 1). In summary, the psychometric properties were obtained for a total of 6 occupational performance measures, which were assessed through 15 articles and 1 manual.

### Included Occupational Performance Measures

The characteristics of the included measures are reported in Table 4. All of the 6 measures were published after 2003. Regarding the purpose of the instrument, 4 measures are used to evaluate children’s perceptions of their competence in performing the activities. The remaining measures, the CAPE and the PAC, are used to identify the participation patterns, perception of enjoyment and preferences in leisure and recreation activities from children’s own perspectives. All of the measures use a Likert response scale as response options to evaluate the perception and preferences. The CAPE additionally reported the use of a dichotomous (i.e., yes or no) rating system and the use of categorical scales for participation patterns. Information on the development and validation of the 6 included occupational performance measures is reported in Table 5.

**Table 4:** Characteristics of the instrument for the assessment of occupational performance.

Instrument (Acronym)	Purpose of instrument	Published year	Number of subscales/ forms	Total number of items	Response options
Perceived Efficacy and Goal Setting System (PEGS) [43, 44]	To enable children with disabilities to set and prioritise their own intervention goals based on their perception regarding competence in performing daily tasks	2004	3 categories	24	1 –a lot like the less competent child; 2 –a little like the less competent child; 3 –a little like the competent child; 4 –a lot like the competent child
Make My Day (MMD) [8]	To collect information regarding young children’s perceptions of their performance, degree of independence and satisfaction from daily activities	2013	10 subscales	34	4-point Likert scale
Children's Assessment of Participation and Enjoyment (CAPE) [45–51]	A measure of children’s participation in recreation and leisure activities as a construct consisting of multiple domains and dimensions	2004	5 sections (diversity, intensity, whom, where, enjoyment). Three levels of scoring: (i) overall participation; (ii) domain scores reflecting participation in formal and informal activities; and (iii) scores reflecting participation in five types of activities (i.e. recreational, active physical, social, skill-based and self-improvement activities)	55	Likert scales are used for each dimension (diversity, intensity, where, with whom, enjoyment) Diversity: Have you done the activity within the last 4 months? Answers “Yes” or “No”. Intensity/Frequency: How often have you performed the activity? Answers from 1- once in the past 4 months, to 7—once a day or more Where: Where do the activities take place? Answers from 1 –at home to 6 –beyond your community. With Whom: With whom do you do the activity? Answers, from 1 –alone to 5 –with others. Enjoyment: (How much do you like or enjoy this activity? Answers from 1- not at all to 5—love it
Preferences for Activities of Children (PAC) [26, 46, 47, 49–51]	Extension of CAPE. Measures child preferences for activities.	2004	As for CAPE	55	A three-point rating ranging from 1 = I would not like to do at all, to 3 = I would really like to do
Child Occupational Self-Assessment (COSA) [52–55]	A self-report of occupational competence and value for activities.	2004 (v 2.0)	2 subscales	24	Competence ratings are given the scores: 1 = I have a big problem doing this; 2 = I have a little problem doing this; 3 = I do this ok; 4 = I am really good at doing this. Values ratings are scored as: 1 = Not really important to me 2 = Important to me 3 = Really important to me 4 = Most important of all to me
Occupational Self-Assessment (OSA) [56, 57]	To capture individuals' perceptions of how illness and disability affect their occupations and rate their own competence and assign value to performance/participation.	2006 (v 2.2)	2 subscales	21	Competence ratings are given the scores: 1 = I have a lot of problem doing this; 2 = I have some difficulty doing this; 3 = I do this well; 4 = I do this extremely well. Values ratings are scored as: 1 = this is not so important to me; 2 = this is important to me; 3 = this is more important to me; 4 = this is most important to me.

**Table 5:** Description of studies and manuals for the development and validation of instrument for the assessment of occupational performance.

Instrument	Reference	Purpose of study	Study population	Age (range[R] and/or Mean[M] Standard deviation[SD])
**PEGS**				
	Vroland-Nordstrom & Krumlinde-Sundholm (I) [43]	To translate, adapt, and assess a Swedish-language version of the PEGS	N = 64; (I) pre-testing: *n* = 19; (II) field testing: *n* = 45; All participants had disabilities	Total sample: R = 5-12y, M = 8.5, SD = 2; (I) R = 5-12y, M = 9y, SD = 3; (II) R = 5-12y, M = 8y, SD = 2
	Vroland-Nordstrom & Krumlinde-Sundholm (II) [44]	Study 1: To evaluate the test–retest reliability of child perceptions using the Swedish version of the PEGS. Study 2: To evaluate agreement between children’s and parents’ perceptions of the child’s competence and choices of intervention goals	N = 44; Test-retest samples: Study 1: *n* = 18; Study 2: *n* = 18; All participants had disabilities	Total sample: R = 5-12y, M = 8y, SD = 2; Study 1: R = 5-12y, M = 8y, SD = 2, Study 2: R = 5-12y, M = 8y, SD = 2
**MMD**				
	Ricon et al. [8]	Internal consistency; Concurrent validity between PEGS and MMD; Hypothesis testing the concurrent validity of the child- and parent-report MMD measures	N = 62; 3 age groups of participants: (I) group 1: *n* = 18; (II) group 2: *n* = 24; (III) group 3: *n* = 20; All participants were TD children	Total sample: R = 4-7y, M = 5.16y, SD = 0.92; (I) R = 4-5y, M = NR, SD = NR; (II) R = 5-6y, M = NR, SD = NR; (III) R = 6-7y, M = NR, SD = NR
**CAPE**				
	Colón et al. [45]	Internal consistency; Hypothesis testing to compare CAPE scores of children with and without disability; To assess the validity and reliability of the newly developed Spanish version	N = 249; (I) 6-10y group: *n* = 126; (II) 11-15y group: *n* = 123; (III) TD: *n* = 198; (IV) developmental disability: *n* = 51; (V) boys: *n* = 126; (VI) girls: *n* = 123; Spanish-speaking children living in Puerto Rico who are either TD or have a developmental disability divided into groups by age, gender, and health status	Total sample: R = 6-15y, M = NR, SD = NR; (I) R = 6-10y, M = NR, SD = NR; (II) R = 11-15y, M = NR, SD = NR; (III) R = 6-15y, M = NR, SD = NR; (IV) R = 6-15y, M = NR, SD = NR; (V) R = 6-15y, M = NR, SD = NR; (VI) R = 6-15y, M = NR, SD = NR
	King et al. [47]	Hypothesis testing to test group differences on CAPE activity type enjoyment scores	N = 427; (I) 6- 8y11m: *n* = 125; (II) 9-11y11m: *n* = 179; (III) 12-15y: *n* = 126; (IV) boys: *n* = 229; (V) girls: *n* = 198; Children with physical functional disabilities born between 1 October 1985 and 30 September 1994 (inclusive)	Total sample: R = 6-15y, M = NR, SD = NR; (I) R = 6- 8y11m, M = NR, SD = NR; (II) R = 9-11y11m, M = NR, SD = NR; (III) 12-15y, M = NR, SD = NR; (IV) R = 6-15y, M = NR, SD = NR; (V) R = 6-15y, M = NR, SD = NR
	Longo et al. [48]	To translate, adapt, and assess the cross-cultural validity and test-retest reliability of a Spanish version of the CAPE; Hypothesis testing to test whether the Spanish version of the CAPE discriminates between TD children and adolescents and those with CP	N = 398; (I) TD: *n* = 199; (II) cerebral palsy (CP): *n* = 199; Children and adolescents (TD and with cerebral palsy but not autism spectrum disorder)	Total sample: R = 8-18y, M = NR, SD = NR; (I) R = 8-18y, M = 13.21y, SD = 3.13; R = 8;0–18;0; (II) R = 8-18y, M = 12.12y, SD = 3.02
	Potvin et al. [50]	To assess the test-retest reliability and content validity of the CAPE for HFA sample	N = 61; (I) Without HFA: *n* = 30; (II) With HFA: *n* = 31; Children with and without high-functioning autism (HFA)	Total sample: R = 7-13y, M = NR, SD = NR; (I) R = 7-13y, M = NR, SD = NR; (II) R = 7-13y, M = NR, SD = NR
	Ullenhag et al. [51]	To translate, adapt, and assess the cross-cultural validity and internal consistency of a Swedish version of the CAPE; Hypothesis testing to compare responses to both the CAPE and Swedish- version CAPE	N = 336; *Cross-cultural group*: (I) TD Swedish children: *n* = 336; *Hypothesis test group;* (II) TD Swedish children: *n* = 336; *Internal consistency groups*: TD Swedish children in 3 age groups: N = 51; (III) youngest age group: *n* = NR; (IV) mid-range age group: *n* = NR; (V) oldest group: *n* = NR	Total sample: R = 6-17y, M = 12y, SD = 2; (I) R = 6-17y, M = 12y, SD = 2; (II) R = 6-17y, M = 12y, SD = 2; (III) R = 6-8y, M = NR, SD = NR; (IV) R = 9-11y, M = NR, SD = NR; (V) R = 12-15y, M = NR, SD = NR
	Nordtorp et al. [49]	To examine the test-retest reliability, measurement error, and internal consistency of the Norwegian version of the CAPE	N = 141; (I) TD: *n* = 107; (II) with disabilities: *n* = 34; Norwegian children and youth, typically developing and with disabilities	Total sample: R = 7-17y, M = NR, SD = NR; (I) R = 7-14y, M = 11.1y, SD = 2.5; (II) R = 8-17y, M = 14.2y, SD = 2.3
	Manual [46]	Internal consistency; Test-retest reliability; Content validity	N = 427; (I) age group 1 (n = 125); (II) age group 2 (n = 176); (III) age group 3 (n = 126); English-speaking children with disabilities. Data also available for family type, family income, number of children living at home, and ethnicity of respondent	Total sample: R = 6-15y, M = NR, SD = NR; (I) R = 6-8y, M = NR, SD = NR; (II) R = 9-11y, M = NR, SD = NR; (III) R = 12-15y, M = NR, SD = NR
**PAC**				
	Manual [46]	Internal consistency; Test-retest reliability; Structural validity; Content validity	N = 427; (I) age group 1 (n = 125); (II) age group 2 (n = 176); (III) age group 3 (n = 126); English-speaking children with disabilities. Data also available for family type, family income, number of children living at home, and ethnicity of respondent	Total sample: R = 6-15y, M = NR, SD = NR; (I) R = 6-8y, M = NR, SD = NR; (II) R = 9-11y, M = NR, SD = NR; (III) R = 12-15y, M = NR, SD = NR
	King et al. [47]	Hypothesis testing to test group differences in PAC activity type preference scores	N = 427; (I) 6- 8y11m: *n* = 125; (II) 9-11y11m: *n* = 179; (III) 12-15y: *n* = 126; (IV) boys: *n* = 229; (V) girls: *n* = 198; Children with physical functional disabilities born between 1 October 1985 and 30 September 1994 (inclusive)	Total sample: R = 6-15y, M = NR, SD = NR; (I) R = 6- 8y11m, M = NR, SD = NR; (II) R = 9-11y11m, M = NR, SD = NR; (III) 12-15y, M = NR, SD = NR; (IV) R = 6-15y, M = NR, SD = NR; (V) R = 6-15y, M = NR, SD = NR
	Nordtorp et al. [49]	To examine the test-retest reliability, measurement error, and internal consistency of the Norwegian version of the PAC	N = 141; (I) TD: *n* = 107; (II) with disabilities: *n* = 34; Norwegian children and youth, typically developing and with disabilities	Total sample: R = 7-17y, M = NR, SD = NR; (I) R = 7-14y, M = 11.1y, SD = 2.5; (II) R = 8-17y, M = 14.2y, SD = 2.3
	Potvin et al. [50]	To assess the test-retest reliability and content validity of the CAPE for HFA sample	N = 61; (I) Without HFA: *n* = 30; (II) With HFA: *n* = 31; Children with and without high-functioning autism (HFA)	Total sample: R = 7-13y, M = NR, SD = NR; (I) R = 7-13y, M = NR, SD = NR; (II) R = 7-13y, M = NR, SD = NR
**COSA**				
	Keller et al. 2005 (I) [52]	To assess the structural validity of the COSA Hypothesis testing to assess whether the COSA discriminates between groups	N = 62; (I) male: *n* = 35; (II) female: *n* = 27; (III) received OT services: *n* = 31; (IV) did not receive OT services: *n* = 31; Children with adequate ability to communicate self-perceptions regarding occupational competence	Total sample: R = 8-17y, M = 11.35y, SD = NR; (I) R = NR, M = NR, SD = NR; (II) R = NR, M = NR, SD = NR; (III) R = NR, M = NR, SD = NR; (IV) R = NR, M = NR, SD = NR
	Keller & Kielhofner 2005 (II) [53]	To assess the structural validity of a refined version of the COSA Hypothesis testing to assess whether the refined COSA’s properties are psychometrically sound	N = 43; Children who are occupational therapy clients	Total sample: R = 8-17y, M = 12.21y, SD = NR
	Kramer et al. 2010 [54]	To assess the structural validity of the COSA; Hypothesis testing to test the external validity of the COSA	N = 502; Child clients of occupational therapist and physical therapist researchers and clinicians internationally	Total sample: R = 6-17y 10m, M = 11y 11.7m, SD = 2 y10.4m
	Romero-Ayuso & Kramer [55]	To assess the internal consistency and cross-cultural validity of the Spanish version of COSA for children with ADHD Hypothesis testing to test whether COSA is an appropriate measure for children with ADHD	N = 30; (I) ADHD diagnosis: *n* = 27; (II) Without ADHD: *n* = 3; Children with ADHD or other disorders associated with attention difficulties	Total sample: R = 7-11y, M = 8.7y, SD = 1.16; (I) R = NR, M = NR, SD = NR; (II) R = NR, M = NR, SD = NR
**OSA**				
	Asgari & Kramer [56]	To assess the structural validity and hypothesis test a second order model of the Persian version of the OSA	N = 336; Students from Tehran junior high schools	Total sample: R = 11-16y, M = 13, SD = 1.2
	Taylor et al. [57]	Hypothesis testing to test the validity of OSA for Adolescents recently diagnosed with acute infectious mononucleosis (MONO) discriminant validity of the OSA to measure for non-recovered and recovered adolescents at 12 m follow-up; Reliability testing	N = 296; (I) non-recovered: *n* = 31; (II) recovered adolescents: *n* = 59; Adolescents recently diagnosed with acute infectious mononucleosis	Total sample: R = 12-18y, M = NR, SD = NR; (I) R = 12-19y, M = NR, SD = NR; (II) R = 12-19y, M = NR, SD = NR

*Notes*. ADHD = Attention deficit hyperactivity disorder; TD = typically developing; R = range; M = Mean; SD = standard deviation; NR = not reported; OT = occupational therapy.

All measures demonstrated some evidence of development and validation although a few included relatively small sample sizes. Of the 6 measures, 4 were developed using children with and without disabilities, 1 using children with disabilities only, and 1 using typically developing children. With regard to the age of participants, 2 measures were developed with children up to 12 years of age (i.e., PEGS and MMD) and the rest with both children and adolescents (6–18 years).

### Psychometric Properties

Table 6 summarises the quality ratings of the psychometric studies of all 6 measures as evaluated against the COSMIN quality criteria. *Hypothesis testing* was the most frequently reported property; all 6 measures had study ratings ranging from fair to excellent quality. This was followed by *cross-cultural validity*; ratings across the 5 measures ranged from poor to excellent quality. Conversely, no measure reported *criterion validity*. The ratings of the quality of the studies of the 4 measures reporting on *internal consistency* and *reliability* ranged from fair to excellent quality. *Structural validity* was reported by 3 measures with ratings of either fair or good quality. The ratings of the studies of the 2 measures reporting on *measurement error* was of excellent quality and *content validity* ranged from poor to excellent quality.

**Table 6:** Overview of the psychometric properties and methodological quality of occupational performance instruments.

Instrument & Author(s)	Year	Internal consistency*	Reliability*	Measurement error*	Content validity*	Structural validity*	Hypotheses testing*	Cross-cultural validity*	Criterion validity*
**MMD**									
Ricon et al. [8]	2013^A^	Fair (42.9)	NR	NR	NR	NR	Fair (29.2)	NR	NR
**PEGS**									
Vroland-Nordstrom & Krumlinde-Sundholm [43, 44]	2012^A^	NR	Excellent (79.5)	NR	NR	NR	Good (56.5)	Excellent (80.5)	NR
**CAPE**									
King et al. [46]	2004^M^	Good (61.9)	Good (70.7)	NR	Good (64.3)	NR	NR	NR	NR
King et al. [47]	2006^A^	NR	NR	NR	NR	NR	Good (74.6)	NR	NR
Colón et al. [45]	2008^A^	Excellent (81.0)	NR	NR	NR	NR	Excellent (86.3)	Good (51.5)	NR
Ullenhag et al. [51]	2012^A^	Excellent (76.2)	NR	NR	Excellent (78.6)	NR	Good (64.8)	Good (54.4)	NR
Potvin et al. [50]	2013^A^	NR	Good (69.0)	NR	Poor (21.4)	NR	NR	NR	NR
Nordtorp et al. [49]	2013^A^	Good (71.5)	Excellent (75.9)	Excellent (82.7)	NR	NR	NR	NR	NR
Longo et al. [48]	2012^A^	NR	Good (72.4)	NR	NR	NR	Good (55.8)	Good (51.5)	NR
**PAC**									NR
King et al. [46]	2004^M^	Good (61.9)	NR	NR	Good (64.3)	Good (58.3)	NR	NR	NR
King et al. [47]	2006^A^	NR	NR	NR	NR	NR	Good (49.7)	NR	NR
Colón et al. [45]	2008^A^	Excellent (81.0)	NR	NR	NR	NR	Excellent (86.3)	Good (51.5)	NR
Potvin et al. [50]	2013^A^	NR	Good (72.4)	NR	Good (71.4)	NR	NR	NR	NR
Nordtorp et al. [49]	2013^A^	Good (71.5)	Good (73.8)	Excellent (82.7)	NR	NR	NR	NR	NR
**COSA**									
Keller et al. (I)[52]	2005^A^	NR	NR	NR	NR	Fair (33.3)	Fair (41.3)	NR	NR
Keller et al. (II)[53]	2005^A^	NR	NR	NR	NR	Fair (50.0)	Fair (41.3)	NR	NR
Romero Ayuso et al. [55]	2009^A^	Fair (38.1)	NR	NR	NR	NR	Poor (17.4)	Poor (24.3)	NR
Kramer et al. [54]	2010^A^	Excellent (90.0)	NR	NR	NR	Good (66.7)	Good (64.8)	NR	NR
**OSA**									
Asgari & Kramer [56]	2008^A^	NR	NR	NR	NR	Good (72.2)	NR	Fair (46.2)	NR
Taylor et al. [57]	2011^A^	NR	Excellent (88.6)	NR	NR	NR	Good (73.9)	NR	NR

Notes.

*The quality of the studies that evaluated the psychometric properties of each instrument were evaluated according to the COSMIN rating. Four-point scale was used (1 = Poor, 2 = Fair, 3 = Good, 4 = Excellent) and the outcome was presented as percentage of rating (Poor = 0–25.0%, Fair = 25.1%-50.0%, Good = 50.1%-75.0%, Excellent = 75.1%-100.0%)

^A^ = Article

^M^ = Manual

NR: not reported.

Table 7 summarises the quality of the psychometric properties of the 6 measures based on the quality criteria described by Terwee et al. [40] (see Table 3). Table 8 provides an overall psychometric quality rating for each of the psychometric properties using the criteria from Schellingerhout et al. [41] (a description of the criteria is provided at the bottom of Table 8). This overall level of evidence score is derived by integrating: 1) the methodological quality of the studies that evaluated the psychometric properties of measures using the COSMIN checklist (Table 6), and 2) the quality criteria for psychometric properties of assessments (Table 7).

**Table 7:** Quality of psychometric properties based on the criteria by Terwee et al. [40].

Assessment Reference	Internal consistency	Reliability	Measurement Error	Content validity	Structural validity	Hypothesis testing	Criterion validity
**MMD**							
Ricon et al. [8]	?	NR	NR	NR	NR	-	NR
**PEGS**							
Vroland-Nordstrom & studyKrumlinde-Sundholm [44]	NR	-	NR	NR	NR	?	NR
**CAPE**							
King et al. [46]	-	-	NR	+	NR	NR	NR
King et al. [47]	NR	NR	NR	NR	NR	+	NR
Colón et al. [45]	?	NR	NR	NR	NR	+	NR
Ullenhag et al. [51]	?	NR	NR	+	NR	+	NR
Potvin et al. [50]	NR	-	NR	NE	NR	NR	NR
Nordtorp et al. [49]	?	-	-	NR	NR	NR	NR
Longo et al. [48]	NR	±	NR	NR	NR	±	NR
**PAC**							
King et al. [46]	+	NR	NR	+	+	NR	NR
King et al. [47]	NR	NR	NR	NR	NR	+	NR
Colón et al. [45]	+	NR	NR	NR	NR	+	NR
Potvin et al. [50]	NR	-	NR	?	NR	NR	NR
Nordtorp et al. [49]	?	+	-	NR	NR	NR	NR
**COSA**							
Keller et al. (I)[52]	NR	NR	NR	NR	+	-	NR
Keller et al. (II)[53]	NR	NR	NR	NR	+	+	NR
Romero Ayuso et al. [55]	?	NR	NR	NR	NR	NE	NR
Kramer et al. [54]	?	NR	NR	NR	+	-	NR
**OSA**							
Asgari & Kramer [56]	NR	NR	NR	NR	?	NR	NR
Taylor et al. [57]	NR	?	NR	NR	NR	+	NR

*Notes*. Quality criteria [40, 41] + = positive rating;? = indeterminate rating;— = negative rating; ± = conflicting data; NR = not reported; NE = not evaluated (study of poor methodological quality according to COSMIN rating—data are excluded from further analyses).

**Table 8:** Overall quality score of assessments for each psychometric property based on levels of evidence by Schellingerhout et al. [41].

Assessment	Internal consistency	Reliability	Measurement Error	Content validity	Structural validity	Hypothesis testing	Criterion validity
**MMD**	Indeterminate	NR	NR	NR	NR	Limited (negative result)	NR
**PEGS**	NR	Strong (negative result)	NR	NR	NR	Indeterminate	NR
**CAPE**	Moderate (negative result)	Strong (negative result)	Strong (negative result)	Strong (positive result)	NR	Strong (positive result)	NR
**PAC**	Strong (positive result)	Conflicting	Strong (negative result)	Moderate (positive result)	Moderate (positive result)	Strong (positive result)	NR
**COSA**	Indeterminate	NR	NR	NR	Moderate (positive result)	Conflicting	NR
**OSA**	NR	Indeterminate	NR	NR	Indeterminate	Moderate (positive result)	NR

*Notes*. Level of Evidence [41]: Strong evidence positive/negative result (consistent findings in multiple studies of good methodological quality OR in one study of excellent methodological quality); Moderate evidence positive/negative results (consistent findings in multiple studies of fair methodological quality OR in one study of good methodological quality); Limited evidence positive/negative result (one study of fair methodological quality); Conflicting findings; Indeterminate = only indeterminate ratings on the measurement property (i.e., score =? in Table 7); NR = not reported.

## Discussion

The purpose of this systematic review was to identify and evaluate the quality of psychometric properties of child-report instruments developed to measure the occupational performance of children. We identified 6 child-report instruments that evaluated a component of occupational performance of children between the ages of 2 and 18 years. Additionally, we systematically searched for and retrieved 15 articles and 1 manual detailing the psychometric properties of the included instruments. This systematic review of child-report measures provides a concise summary of the current selection of psychometric properties of these measures. The COSMIN framework was employed to guide a comprehensive summary of the psychometric properties of six instruments [34]. The application of the COSMIN checklist-based taxonomy allowed for a critical evaluation of the quality and extent of psychometric evidence of the 15 research articles and 1manual on the 6 child-report occupational performance instruments [33, 34]. Responsiveness was outside the scope of the current review.

### Quality of the Studies using the COSMIN Taxonomy

The COSMIN checklist provides information about the quality of the studies that examined the measures' psychometric properties [33, 34]. In regards to reliability, *internal consistency* was detailed for half of the measures (CAPE, PAC, COSA), whilst four of the measures detailed reliability testing (CAPE, PAC, PEGS, OSA). This review indicated good to excellent study quality for both *internal consistency* as well as *reliability* for the majority of the measures, except for the MMD and COSA (one study) which received a fair rating for internal consistency.

Only two of the six measures (CAPE, PAC) reported *measurement error*. Both these measures received an excellent score for study quality. Consequently, the assessment of study quality of reliability for both the CAPE and the PAC is comprehensive, as these measures included psychometric properties for *internal consistency*, *reliability*, *and measurement error*; no other measures included properties for these three elements. Considering the lack of all three psychometric properties being reported, a true indication of overall *reliability* for four measurements (PEGS, MMD, COSA, OSA) is not possible. Consideration of the *measurement error* is essential when selecting outcome measures for a study, as low error allows the measure to be used to detect smaller treatment effects. A low *measurement error* in relation to its minimal important change (MIC) means that clinical trials require smaller sample sizes than measures where the opposite applies [58]. Therefore, future studies of the PEGS, MMD, COSA, and OSA should attempt to gain a more comprehensive picture of the psychometric properties relating to reliability by including assessment of *internal consistency*, *reliability* and *measurement error*.

Within the COSMIN taxonomy, *construct validity* consists of *content validity*, *structural validity* and *hypothesis testing* [33, 34]. Detailing these components of construct validity is important, and a lack of reporting can have implications in clinical practice. For instance, when a scale or measure is used without the documented measurement properties (such as *construct validity*) potential negative consequences can occur, such as an error in clinical judgment or the inaccurate interpretation of assessment results by practitioners. It is crucial that practitioners are able to investigate how well a measure assesses what it claims, as well as how well it holds its meaning across varied contexts and sample groups for confident use within clinical settings. The PAC was the only incorporated measure to include all three elements of *construct validity*, with ratings of study quality ranging between good and excellent quality found for each element. The PEGS, MMD, COSA, and OSA did not provide any evidence of content validity, highlighting a need for further research of the psychometric properties of these instruments.

This review also revealed that the PEGS, CAPE, and MMD did not have any published information in the domain of *structural validity*; emphasising again a need for further research. For *hypothesis testing*, the majority of the measures (PEGS, CAPE, PAC, OSA) provided evidence of conducting studies at a good or excellent level of quality, whilst the COSA and MMD had evidence of studies of poor or fair level of quality. Taken together, the COSA and the MMD may need a revision of the measures to improve evidence that they have sound construct validity.

*Cross-cultural validity* was reported for all measures except the MMD with variability in quality of studies ranging from poor (COSA) to excellent (PEGS). This indicates that four of the measures were adequately (PEGS, CAPE, PAC) translated or culturally adapted from the original version, whilst the translated or culturally adapted COSA and OSA were limited in their study quality. It is important to note that none of the six measures reported *criterion validity*. Thus, comparisons between these measures and a “gold standard” measure of occupational performance could not be made. However, as there is no widely accepted gold standard of assessment for the occupational performance of children, it is no surprise that we were unable to recover evidence of *criterion validity*.

### Overall Quality of Psychometric Properties

Varying results were found for overall quality of measurement properties using the level of evidence criteria by Schellingerhout et al. [41]. The occupational performance self-report measure with the most robust psychometric properties to date was the PAC, given that 7 of the 8 psychometric properties were evaluated, with overall quality ratings of moderate to strong with positive results for four psychometric properties. Evidence for *reliability* was however found to be conflicting between studies, with strong negative results for *measurement error*. CAPE had five scores of moderate to strong quality, however produced negative ratings for its *reliability* and *measurement error*. The measures with the least evidence in terms of sound psychometric properties was the PEGS and MMD with ratings of indeterminate, limited poor and strong negative results. Interestingly, despite the studies investigating the reliability of the PEGS and CAPE rated as strong for methodological quality, negative results were found in terms of psychometric quality criteria. The same pattern was evident for *measurement error* for the CAPE and PAC. Four of the six assessments have psychometric properties of indeterminate overall quality due to not reporting on statistical analyses, such as factor analysis or having a doubtful design. Both the COSA and OSA assessments were rated as only having one psychometric property with moderate positive evidence, with both receiving ratings of indeterminate or conflicting levels of evidence. These findings highlight the need for further, rigorous testing of the properties of these measures before they may be deemed as being psychometrically sound.

The results of the current systematic review also revealed that a number of child-report measures of occupational measures were validated with modest sample sizes and/or developed with small sample sizes (< 300 children). For example, the MMD was developed and validated using a total sample size of 62 children [8]. The other measure developed and/or validated with small sample sizes was the PEGS [43, 44]. Validation studies which use a limited sample size are not reliable for reaching conclusions about the psychometric properties of a measure, as the small number of participants may not be generalisable to a wider population. This can result in ill-informed clinical assessment. Thus, future studies of the COSA, MMD, and PEGS using large numbers in a normative sample are needed in order to increase the generalisability of the results of these measures to the general population. This will allow clinicians to make better informed assessments of children’s occupational performance.

### Limitations

Whilst this systematic review aimed to be rigorous, there were a few limitations. Information published in languages other than English were not included, thus, some relevant findings regarding the psychometric properties of child-report occupational performance may have been excluded. Furthermore, we did not contact all authors who published research on the psychometric properties of occupational performance measures directly, so some information may have been overlooked. Evaluating the quality of responsiveness as a psychometric property was outside the scope of this systematic review. Future studies could assess the responsiveness of child-report measures to change in occupational performance.

## Conclusion

As occupational performance is central to the practice of occupational therapy, it is important to use sound measures in practice in order to provide measures with excellent psychometric quality to accurately assess and treat clients. The current systematic review reported the results of 15 studies and one manual reporting on evidence of the psychometric properties of six child-report measures of occupational performance for children. In order to consistently rate the reliability and validity information reported about the measures, the COSMIN taxonomy was used. Whilst the majority of instruments had conducted good quality studies to evaluate the psychometric properties of measures (PEGS, CAPE, PAC, OSA), the quality of the studies for two of these measures was relatively weak (MMD, COSA). When integrating the quality of the reported psychometric properties with the quality of the studies, only the PAC stood out as having superior psychometric qualities. These findings are concerning given that these measures are used routinely in clinical practice in the assessment and treatment of children. Thus, this review highlights the need for more research examining the psychometric properties of child-report measures of occupational performance, and an improvement of the psychometric properties of existing measures using sound techniques.

## Supporting Information

S1 Table PRISMA checklist for the current review. From: Moher D, Liberati A, Tetzlaff J, Altman DG, The PRISMA Group (2009). Preferred Reporting Items for Systematic Reviews and Meta-Analyses: The PRISMA Statement. PLoS Med 6(6): e1000097. doi:10.1371/journal.pmed1000097.
(DOC)
